# Changes in serum and synovial fluid biomarkers after acute injury (NCT00332254)

**DOI:** 10.1186/ar3216

**Published:** 2010-12-31

**Authors:** Jonathan B Catterall, Thomas V Stabler, Carl R Flannery, Virginia B Kraus

**Affiliations:** 1 Division of Rheumatology, Department of Medicine, Duke University, Genome Science Research Building I, 905 South LaSalle Street, Durham, NC 27710, USA; 2 Tissue Repair Research Unit, BioTherapeutics Division, Pfizer Inc., 200 Cambridge Park Drive, Cambridge, MA 02140, USA

## Abstract

**Introduction:**

Acute trauma involving the anterior cruciate ligament is believed to be a major risk factor for the development of post-traumatic osteoarthritis 10 to 20 years post-injury. In this study, to better understand the early biological changes which occur after acute injury, we investigated synovial fluid and serum biomarkers.

**Methods:**

We collected serum from 11 patients without pre-existing osteoarthritis from a pilot intervention trial (5 placebo and 6 drug treated) using an intra-articular interleukin-1 receptor antagonist (IL-1Ra) therapy, 9 of which also supplied matched synovial fluid samples at presentation to the clinic after acute knee injury (mean 15.2 ± 7.2 days) and at the follow-up visit for reconstructive surgery (mean 47.6 ± 12.4 days). To exclude patients with pre-existing osteoarthritis (OA), the study was limited to individuals younger than 40 years of age (mean 23 ± 3.5) with no prior history of joint symptoms or trauma. We profiled a total of 21 biomarkers; 20 biomarkers in synovial fluid and 13 in serum with 12 biomarkers measured in both fluids. Biomarkers analyzed in this study were found to be independent of treatment (*P *> 0.05) as measured by Mann-Whitney and two-way ANOVA.

**Results:**

We observed significant decreases in synovial fluid (sf) biomarker concentrations from baseline to follow-up for _sf_C-Reactive protein (CRP) (*P *= 0.039), _sf_lubricin (*P *= 0.008) and the proteoglycan biomarkers: _sf_Glycosaminoglycan (GAG) (*P *= 0.019), and _sf_Alanine-Arginine-Glycine-Serine (ARGS) aggrecan (*P *= 0.004). In contrast, we observed significant increases in the collagen biomarkers: _sf_C-terminal crosslinked telopeptide type II collagen (CTxII) (*P *= 0.012), _sf_C1,2C (*P *= 0.039), _sf_C-terminal crosslinked telopeptide type I collagen (CTxI) (*P *= 0.004), and _sf_N-terminal telopeptides of type I collagen (NTx) (*P *= 0.008). The concentrations of seven biomarkers were significantly higher in synovial fluid than serum suggesting release from the signal knee: IL-1β (*P *< 0.0001), fetal aggrecan FA846 (*P *= 0.0001), CTxI (*P *= 0.0002), NTx (*P *= 0.012), osteocalcin (*P *= 0.012), Cartilage oligomeric matrix protein (COMP) (*P *= 0.0001) and matrix metalloproteinase (MMP)-3 (*P *= 0.0001). For these seven biomarkers we found significant correlations between the serum and synovial fluid concentrations for only CTxI (*P *= 0.0002), NTx (*P *< 0.0001), osteocalcin (*P *= 0.0002) and MMP-3 (*P *= 0.038).

**Conclusions:**

These data strongly suggest that the biology after acute injury reflects that seen in cartilage explant models stimulated with pro-inflammatory cytokines, which are characterized by an initial wave of proteoglycan loss followed by subsequent collagen loss. As the rise of collagen biomarkers in synovial fluid occurs within the first month after injury, and as collagen loss is thought to be irreversible, very early treatment with agents to either reduce inflammation and/or reduce collagen loss may have the potential to reduce the onset of future post-traumatic osteoarthritis.

**Trial registration:**

The samples used in this study were derived from a clinical trial NCT00332254 registered with ClinicalTrial.gov.

## Introduction

Acute trauma to the anterior cruciate ligament (ACL) or meniscus has recently been demonstrated to be a major risk factor for the development of osteoarthritis (OA) with a 50% chance of a patient developing symptomatic OA 10 to 20 years post-injury repair [1]. ACL injuries represent a quarter of all knee injuries with an annual incidence of at least 81 per 100,000 population which equates to at least 246,000 ACL injuries and more than 175,000 reconstructions in the US annually among persons aged 10 to 64 years old [2]. Knee injury is a leading cause of OA in young people, with young women having a three- to five-fold higher risk of an ACL injury than young men [1,3]. A prospective study of young adults revealed a 14% cumulative incidence of self-reported knee OA by the age of 65 years for those who had any knee injury during their adolescence or young adulthood compared with a 6% incidence for those without a history of knee joint injury [4]. Post-traumatic OA is believed to account for up to 5.6 million per year or 12% of the total US cases of symptomatic OA with an estimated cost in 2006 of $3.06 billion per year or 0.15% of total US direct health care costs [5,6]. It is increasingly believed that the development of later OA in the injured joints is initiated by both bone and cartilage damage caused during the initial traumatic and inflammatory event coupled with possible long term changes to joint loading. After injury, it is proposed that abnormal joint motion and loading lead to a biological response dominated by catabolic activity [1,7]. However, unlike primary OA, post-traumatic secondary OA is initiated by intra-articular pathogenic processes with a known date of onset, namely the date of joint injury. This makes it much more amenable to early intervention than primary OA whose onset is not clearly definable at this time.

Following acute trauma, an initial flare of cytokines has been reported which mirrors that seen in wound healing and includes tumor necrosis factor (TNF)α, interleukin (IL)-1β, IL-6, IL-8, IL-1 receptor antagonist (IL-1Ra) and IL-10 [8]. Cartilage damage has been demonstrated by the release of proteoglycan and collagen fragments after ACL rupture [9,10]; release is maximal in the first few weeks but can persist at a significantly elevated concentration in synovial fluid for decades following injury [10-15]. Other matrix molecules such as matrix metalloproteinase (MMP)-3, tissue inhibitor of metalloproteinase (TIMP)-1 and cartilage oligomeric matrix protein (COMP) have also been demonstrated to have persistently elevated concentrations in synovial fluid after ACL injury [15,16] The increased matrix turnover may not be limited to merely the affected knee as there is evidence to suggest that the concentrations of aggrecan, COMP and MMP-3 are also elevated in the uninjured contralateral knee of ACL rupture patients [17], possibly as a consequence of altered loading. A canine ACL rupture model of OA also demonstrates increased synthesis and turnover of proteoglycan in early arthritis [18,19]. Thus, biomarker profiles could potentially indicate risk for progression to OA based on the identities, quantities, and temporal patterns of expression of specific joint tissue components. In this study we investigated a wide range of synovial fluid and serum biochemical biomarkers, at two time points, to better understand the changes and damage which occur to both the bone and cartilage early in the course of severe knee trauma. Many biomarkers are not specific for the joint and there is a possibility that the concentrations of a particular biomarker, when measured in the serum, could be affected by release from alternative sites due to increased systemic inflammation following acute injury. Therefore, in this study we also investigated the relationship between the serum and synovial fluid concentrations of individual biomarkers to determine whether measurements made in the serum reflect the concentrations observed within the signal knee.

## Materials and methods

### Sample collection

All samples were collected with informed consent and this research was performed with the approval of the Duke University Investigational Review Board and was carried out in compliance with the Helsinki Declaration. Synovial fluid and matched serum samples were collected at baseline (within four weeks of injury) and at follow-up at the time of arthroscopic repair surgery. Synovial fluid was centrifuged (8°C, 3,500 g, five minutes), and the supernatant aliquoted and frozen at -80°C within two hours of collection. Serum was also aliquoted and frozen at -80°C until analysis.

### Patient characteristics

Samples for this study were derived from a randomized double-blinded placebo-controlled pilot trial of a single injection of the short-acting intra-articular IL-1Ra therapy, anakinra, administered at the time of presentation to the clinic [20]. The samples used in this study were derived from a clinical trial, NCT00332254 registered with ClinicalTrial.gov. The trial consisted of 11 patients, 5 saline injected placebo and 6 anakinra injected study participants. We collected serum from 11 patients, 9 of which also supplied matched synovial fluid samples (4 placebo and 5 drug), who presented to the Duke Sports Medicine clinic with a history of recent (within the previous month) severe knee injury due to sports injury. The cohort was young and otherwise healthy with a mean age at enrollment of 23 ± 3.5 years and a 6/5 male/female split. The study was limited to individuals younger than 40 years of age with no prior history of joint symptoms or trauma to exclude patients with pre-existing OA. Patients were enrolled as soon after initial injury as possible with a mean baseline enrollment time of 15.2 ± 7.2 days after injury and a mean follow-up time of 47.6 ± 12.4 days after injury with samples collected at time of surgery. The joint pathology was defined by clinical knee magnetic resonance images obtained prior to the baseline assessments, and included, in addition to evidence of ACL tear in all patients, other knee joint tissue damage including bone contusions, medial collateral ligament tears, meniscal tears and chondral defects. All biomarkers within this report were found to be independent of treatment by both two-way Analysis of Variance (ANOVA) and by Mann-Whitney tests, so for the purposes of statistical analysis, we combined the drug and control groups to improve statistical power.

### Biomarker analyses

We investigated a range of biomarkers summarized in Table 1. The majority of biomarker measurements were obtainable with commercially available reagents and assays were performed as per the manufacturer's instructions (details in Table 1). The remaining biomarker assays utilized methods that have been previously described and summarized briefly below.

**Table 1:** Summary and sources of the assays used in this study

Analyte	Details	Assay (Reference)	Sample	Supplier
IL-1β	Inflammatory Cytokine	ELISA [8]	SF, Serum	R&D Systems, Minneapolis, MN
hsCRP	Inflammatory marker	ELISA [43]	SF, Serum	United Biotech, Inc, Mountain View, CA
Sulphated GAG	Total proteoglycan loss (mainly aggrecan)	Chemical assay	SF	Kamiya Biomedical Company, Seattle, WA
ARGS-aggrecan	Aggrecan cleavage assay	ELISA [21]	SF	Pfizer In house
FA846	Fetal aggrecan matrix protein (CS846 epitope)	ELISA	SF, Serum	IBEX, Montreal, Quebec, Canada
CS846	Aggrecan epitope	Competitive ELISA [43]	Serum	IBEX, Montreal, Quebec, Canada
C2C	Collagenase neoepitope of Collagen II	ELISA [43]	SF, Serum	IBEX, Montreal, Quebec, Canada
CTxII	Collagen II degradation marker	ELISA [43]	SF	Nordic Bioscience, Herlev, Denmark
C1,2C	Type I/II collagen degradation	ELISA [43]	SF, Serum	Nordic Bioscience, Herlev, Denmark
CTxI	Type I collagen degradation	ELISA [44]	SF, Serum	Osteometer MediTech, Inc, Hawthorene, CA
NTX	Bone formation marker	ELISA [45]	SF, Urine	Osteomark, Princeton, NJ
CPII	Collagen II synthesis marker	ELISA [43]	SF, Serum	IBEX, Montreal, Quebec, Canada
Osteocalcin	Bone synthesis marker	ELISA [43]	SF, Serum	Quidel Corporation, San Diego, CA
β-Aspartate	Age dependant protein modification	Enzyme Assay [46]	SF, Serum	Promega, Madison, WI
D-Asx	Age dependant protein modification	HPLC [22]	SF, Serum	Kraus laboratory In house
D-Serine	Age dependant protein modification	HPLC [22]	SF, Serum	Kraus laboratory In house
sCD44	Cartilage marker	ELISA [47]	SF, Serum	eBioscience, Inc, San Diego, CA
COMP	Cartilage matrix protein	ELISA [23]	SF, Serum	Kraus laboratory
Tenascin C	Cartilage matrix protein	ELISA [48]	SF	IBL International GmbH, Toronto, ON, Canada
Lubricin	Cartilage matrix protein	ELISA	SF	Pfizer In house
MMP-3	Proteolytic enzyme	ELISA [49]	SF, Serum	R&D Systems, Inc., Minneapolis, Mn

COMP, cartilage oligomeric matrix protein; CRP, C-reactive protein; GAG, glycosaminoglycan.

### sGAG determination using alcian blue

A commercial sGAG kit was used as per the manufacturer's instructions (Kamiya Biomedical Company, Seattle, WA, USA). Briefly, 50 μl of each sample (either standard or synovial fluid) was adjusted to 4 M guanidine-HCl by addition of an equal volume of 8 M guanidine-HCl, mixed and incubated at room temperature (RT) for 15 minutes. A further 50 μl of a 0.3% volume/volume (v/v) H_2_SO_4 _and 0.75% v/v Trition X-100 solution was added, mixed and incubated at RT for 15 minutes. After incubation, 750 μl of alcian blue solution (0.03 M Guanidine-HCl, 0.1% v/v H_2_SO_4_, 0.25% v/v triton X-100) was added, mixed and incubated for 15 minutes at RT before centrifugation at 12,000 g for a further 15 minutes. The supernatant was discarded and 500 μl of dimethyl sulfoxide solution (40% v/v DMSO, 0.05 M MgCl_2_) added and mixed for 15 minutes at RT before centrifugation at 12,000 g for 15 minutes. The supernatant was discarded and the pellet redissolved in Gu-Prop solution (4 M Guanidine-HCl, 33% v/v 1-propanol, 0.25% v/v triton X-100) for 15 minutes on a shaker. Samples were read at 595 nm and the GAG level determined from a linear standard curve (12.5 to 400 μg/ml).

### Alanine-Arginine-Glycine-Serine (ARGS)-aggrecan ELISA

Synovial fluid samples were adjusted to contain 50 mM Tris Acetate, 0.1 M NaCl and 5 mM CaCl_2_, pH 7.3, and 1× protease inhibitor cocktail I (Calbiochem/EMD Chemicals, Gibbstown, NJ, USA). Samples were deglycosylated by treating with 0.2 U/ml chondroitinase ABC, 0.2 U/ml keratanase, and 0.02 U/ml keratanase II (Seikagaku America, Falmouth, MA, USA) at 37°C for two to three hours. Sample dilutions were then applied to microtiter plate wells coated with anti-human aggrecan mAb AHP0022 (Invitrogen, Carlsbad, CA, USA) and incubated for two hours at RT. After washing, horse radish peroxidase (HRP)-labeled anti-ARGS monoclonal antibody (mAb) BC-3 (Abcam, Cambridge, UK) was added to the wells, followed by incubation for 1.5 hours at RT, washing, and detection with 3,3', 5,5"-tetramethylbenzidine (TMB) One Component HRP substrate (BioFX Laboratories, Owings Mills, MD, USA). The concentration of ARGS-aggrecan for each sample was determined based on a standard curve generated using ADAMTS4-digested human aggrecan as previously described [21].

### FA-846 fetal aggrecan sandwich ELISA

FA-846 aggrecan was measured as per the manufacturer's (IBEX, Montreal, QC, Canada) instructions. Briefly, 50 μl of assay buffer and either 50 μl of diluted sample or standards were added to each well of a pre-coated plate and incubated at RT for 1.5 hours with shaking (600 to 700 rpm). The plate was washed six times using the supplied wash buffer before addition of 100 μl of biotinylated FA-846 antibody (50 μl of stock diluted in the supplied buffer II) and incubated with shaking for 30 minutes at RT. Excess antibody was removed with a further six washes with the supplied wash buffer before the addition of 100 μl of 5 pg/ml streptavidin-HRP diluted in buffer II and incubation for 30 minutes at RT with shaking. Excess streptavidin-HRP was removed with six washes before the addition of 100 μl/well of TMB reagent. Once developed, the TMB reaction was stopped using 100 μl/well of stop solution and read at 450 nm.

### Quantification of racemized amino acids

Racemized Asx (Aspartate and Aspargine) and Serine concentrations in synovial fluid were quantified using a previously described high performance liquid chromatography (HPLC) approach developed and validated in our laboratory [22]. Briefly, purified proteins were acid hydrolyzed in 6 M HCl at 105°C for 8 h. The resulting free D- and L-amino acids were derivatized to fluorescent compounds by addition of o-phthaldialdehyde and N-tert-butyloxycarbonyl-L-cysteine. The subsequent derivatives were separated by reversed-phase HPLC using a C18 column and mobile phases of 0.2 M acetic acid adjusted to a pH of 6.0 and an acetonitrile gradient. The resulting peaks were quantified fluorometrically (ex340 nm, em440 nm) by comparison to commercially available pure D- and L-amino acids (Sigma-Aldrich, St. Louis, MO, USA).

### COMP ELISA

Serum and synovial fluid COMP were quantified by ELISA using the mAb 16F12 for capture and biotinylated mAb 17C10 for detection as previously described [23] with the following minor modifications. Plates were blocked with 3% BSA/PBS/0.02% sodium azide for two hours. Synovial fluid was diluted 1:400 and 1:800. Streptavidin-alkaline phosphatase conjugate (ExtrAvadin, Sigma, St. Louis, MO, USA) was diluted 30,000 × in 0.01% BSA/PBS/0.02% sodium azide and the phosphatase substrate (4-nitrophenyl phosphate disodium salt hexahydrate, Sigma) was used as the detection agent. Plates were read at wavelength 405 nm after 20 minutes of incubation.

### Lubricin ELISA

Synovial fluid samples were deglycosylated as described for the ARGS-aggrecan ELISA before sample dilutions were applied to microtiter plate wells coated with an anti-lubricin mAb (Clone 5; Pfizer, Cambridge, MA, USA) and incubated for one hour at RT. After washing, a second anti-lubricin mAb (HRP-lableled Clone 26; Pfizer) was added to the wells, followed by incubation for one hour at RT, washing, and detection with TMB One Component HRP substrate (BioFX Laboratories, Owings Mills, MD, USA). The concentration of lubricin for each sample was determined based on a standard curve generated using a recombinant human lubricin protein construct, LUB1 [24].

### Statistical methods

Statistical analyses were performed using GraphPad Prism version 4.02 (GraphPad Software, Inc, La Jolla, CA, USA). Due to the small sample size used in this study it was not realistic to assume a Gaussian distribution and so all statistical tests used were non-parametric: for all correlations Spearman Rank correlation was used; for paired data Wilcoxon Signed Rank test was used. Samples for this study were derived from a pilot trial of a single injection, intra-articular anti-IL-1 therapy [20] and as described above, the drug and control groups were combined for purposes of statistical analysis.

## Results

### Biomarker concentrations in the setting of acute knee injury

To gain an understanding of the effect of joint injury on joint tissue turnover and pathology, we investigated 20 biomarkers in synovial fluid and 13 in serum soon after acute knee injury and after a period of recovery but prior to surgery.

Initially we compared the baseline and follow-up data for each biomarker to determine whether there were any significant changes in biomarker concentrations between the two time points (Table 2). In synovial fluid (_sf_), we observed a significant decrease between the two collection time points for the inflammatory marker _sf_CRP (*P *= 0.039), the cartilage superficial zone protein _sf_lubricin (*P *= 0.008) and the biomarkers of proteoglycan: _sf_GAG (*P *= 0.019) and the aggrecanase cleaved aggrecan marker _sf_ARGS aggrecan (*P *= 0.004). We observed decreasing trends for the remaining inflammation and joint tissue biomarkers, _sf_IL-1β (*P *= 0.124), fetal aggrecan _sf_FA846 (*P *= 0.074) and _sf_COMP (*P *= 0.055). In contrast, we observed significant increases between the two time points for several of the synovial fluid biomarkers of collagen turnover: C-terminal crosslinked telopeptide type II collagen (_sf_CTxII) (*P *= 0.012), _sf_C1,2C (*P *= 0.039), C-terminal crosslinked telopeptide type I collagen (_sf_CTxI) (p = 0.004) and N-terminal telopeptides of type I collagen (_sf_NTx) (*P *= 0.008). While we observed small increases in the collagen degradation marker _sf_C2C, the collagen synthesis marker _sf_CPII, and the bone synthesis marker _sf_Osteocalcin, these were not significant and may reflect the limitation of our small sample size. Finally we observed an increasing trend for the biomarker of protein age, D-Serine (*P *= 0.055).

**Table 2:** Biomarker concentrations at baseline and follow-up in serum and synovial fluid after acute knee injury

	Synovial Fluid Levels			Serum Levels			Serum vs Synovial Fluid	
			
Biomarker	Baseline Mean ±SD	Follow Up Mean ±SD	*P-value*	Baseline Mean ±SD	Follow Up Mean ±SD	*P-value*	Mean Ratio (synovial fluid/serum levels) ± SD, *P*-value	Correlation (between serum and synovial fluid levels), *P*-value
*Inflammation markers*								
IL-1β (pg/ml)	1.02 ± 0.66	0.61 ± 0.22	*P = 0.124*	0.17 ± 0.06	0.24 ± 0.12	*P = 0.102*	↑*4.3 ± 2.1, p < 0.0001*	r_s _= 0.358, *P *= 0.123
CRP (μg/ml)	1.67 ± 1.28	0.68 ± 0.64	*P = 0.039*	3.18 ± 2.93	1.78 ± 2.10	*P = 0.102*	↓*3.5 ± 4.2, p = 0.012*	*r*_*s *_*= 0.658, P = 0.002*
*Proteoglycan markers*								
GAG (μg/ml)	330.7 ± 203.2	170.2 ± 45.9	*P = 0.008*	---	---	---	---	---
ARGS aggrecan (μg/ml)	230.7 ± 236.7	63.2 ± 54.0	*P = 0.004*	---	---	---	---	---
FA846 (ng/ml)	1134.0 ± 158.1	886.5 ± 295.8	*P = 0.074*	31.2 ± 35.1	32.4 ± 43.8	*P = 0.520*	↑*59.1 ± 6.3, p = 0.0001*	*r*_*s *_*= -0.179, P = 0.464*
CS846 (ng/ml)*	---	---	---	72.3 ± 139.2	58.1 ± 131.6	*P = 0.413*	---	---
*Collagen markers*								
C2C (ng/ml)	184.1 ± 37.6	208.9 ± 56.3	*P = 0.301*	226.5 ± 34.8	216.3 ± 49.2	*P = 0.700*	↓*1.2 ± 0.5, p = 0.232*	r_s _= -0.463, *P *= 0.046
CTxII (μg/L)	0.59 ± 0.58	1.09 ± 0.63	*P = 0.012*	---	---	---	---	---
C1,2C (μg/ml)	0.28 ± 0.10	0.37 ± 0.08	*P = 0.039*	0.53 ± 0.18	0.51 ± 0.19	*P = 0.638*	↓*1.8 ± 1.2, p = 0.005*	r_s _= 0.209, *P *= 0.391
CTxI (ng/ml)	0.75 ± 0.39	1.23 ± 0.67	*P = 0.004*	0.52 ± 0.32	0.66 ± 0.31	*P = 0.083*	↑*1.6 ± 0.5, p = 0.0002*	r_s _= 0.819, *P *< 0.0001
NTx (nM BCE)	15.58 ± 4.37	19.64 ± 7.78	*P = 0.008*	18.67 ± 6.92	21.6 ± 4.2	*P = 0.054*	↑*1.2 ± 0.2, p = 0.012*	r_s _= 0.828, *P *< 0.0001
CPII (ng/ml)	308.1 ± 257.4	318.3 ± 185.1	*P = 0.820*	365.2 ± 181.5	288.2 ±106.8	*P = 0.102*	↑*1.3 ± 0.9, p = 0.976*	r_s _= -0.102, *P *= 0.679
Osteocalcin (ng/ml)	15.43 ± 4.99	17.08 ± 6.16	*P = 0.301*	19.91 ± 8.54	16.3 ± 5.6	*P = 0.032*	↑*1.2 ± 0.3, p = 0.012*	r_s _= 0.747, *P *= 0.0002
*Post-translational markers of protein age*								
β-Aspartate (μM)	0.94 ± 0.32	0.76 ± 0.37	*P = 0.129*	---	---	---	---	---
D-Asx (D/D+L)	0.018 ± 0.001	0.019 ± 0.002	*P = 0.423*	---	---	---	---	---
D-Serine (D/D+L)	0.002 ± 0.001	0.003 ± 0.001	*P = 0.055*	---	---	---	---	---
*Other Biomarkers*								
sCD44 (ng/ml)*	128.1 ± 49.0	105.0 ± 28.2	*P = 0.250*	115.9 ± 26.9	110.7 ± 39.0	*P = 0.820*	↑*1.1 ± 0.4, p = 0.922*	r_s _= 0.301, *P *= 0.226
COMP (μg/ml)	140.9 ± 65.4	99.1 ± 32.3	*P = 0.055*	8.6 ± 4.1	8.0 ± 4.4	*P = 0.278*	↑*21.2 ± 15.3, P = 0.0001*	r_s _= -0.326, *P *= 0.173
Tenascin C (ng/ml)	813.2 ± 428.6	908 ± 599	*P = 0.570*	---	---	---	---	---
Lubricin (nM equ)	390.4 ± 159.6	215.9 ± 59.0	*P = 0.008*	---	---	---	---	---
MMP-3 (ng/ml)	29.02 ± 20.84	30.89 ± 26.56	*P *= 0.910	0.05 ± 0.02	0.04 ± 0.02	*P *= 0.148	↑738.8 ± 602.5, *P *= 0.0001	r_s _= 0.479, *P *= 0.038

Results represent mean ± SD data for *n *= 9 patients for synovial fluid values and for serum *n *= 11 or *n *= 9 for * marked biomarkers due to sample depletion. Non-parametric paired analyses by Wilcoxon Signed Rank test were performed to evaluate for significant differences between baseline and follow up time points and between serum and synovial fluid values. Non parametric Spearman Rank correlations (*r*_*s*_) were performed to determine correlations between synovial fluid and serum and the ratios reported are mean ratios ± SD.

In the serum (_s_) we observed fewer significant changes, with only _s_Osteocalcin (*P *= 0.032) demonstrating a significant decrease between the two time points, while both CTxI (*P *= 0.083) and NTx (*P *= 0.054) showed increasing trends with time, which were consistent with the observations in synovial fluid. We also used the complementary approach of Spearman Rank Correlation to analyze time from injury and synovial fluid biomarker concentration. Interestingly, we observed significant decreases with time for _sf_CRP (*P *= 0.041, r_s _= -0.473), _sf_GAG (*P *= 0.027, r_s _= -0.506), _sf_ARGS aggrecan (*P *= 0.003, r_s _= -0.638), _sf_β-Aspartate (*P *= 0.025, r_s _= -0.525) and _sf_lubricin (*P *= 0.027, r_s _= -0.506), while the collagen markers _sf_CTxII (P = 0.002, r_s _= 0.659) and _sf_C1,2C (*P *= 0.005, r_s _= 0.613) demonstrated significant increases with time.

### Comparison of biomarkers in matched serum and synovial fluid

To investigate whether the biomarker values measured in serum behaved as surrogates for the concentrations found within the signal knee after acute injury, we compared biomarker concentrations in synovial fluid and serum from the matched samples collected from the same patients. Due to assay sensitivity and specificity, we were able to measure 12 of the 20 synovial fluid biomarkers in matched serum and synovial fluid samples (Table 2). We hypothesized that biomarkers produced in the injured signal knee would have significantly higher concentrations in the synovial fluid than in the circulating serum. When we compared the concentrations of the 12 biomarkers measured in both fluids, seven demonstrated significantly higher concentrations in the synovial fluid than serum: IL-1β (*P *< 0.0001), FA846 (*P *= 0.0001), CTxI (*P *= 0.0002), NTx (*P *= 0.012), Osteocalcin (*P *= 0.012), COMP (*P *= 0.0001) and MMP-3 (*P *= 0.0001). The biggest differences between synovial fluid and serum were observed for MMP-3 and COMP with fold differences of 738.8 and 21.2 respectively. Smaller fold differences were observed for FA846 (5.9×), IL-1β (4.3×), CTxI (1.6×), NTx (1.2×) and osteocalcin (1.2×). The inflammatory marker CRP, produced in the liver, showed significantly higher concentrations (*P *= 0.012) in the serum than in synovial fluid (3.5×). It is of note that CRP is not an OA specific marker, it is an acute phase protein and upregulated in many different conditions, which limits its specificity. We also observed significantly higher concentrations of C1,2C (*P *= 0.005) in the serum suggesting a systemic source or rapid clearance from the joint for this biomarker (1.8×).

To further investigate the utility of the serum biomarkers to reflect signal knee synovial fluid concentrations, we performed Spearman Rank correlations between the matched synovial fluid and serum samples. Of the seven biomarkers with significantly higher concentrations in synovial fluid than serum, the concentrations of four biomarkers were found to correlate between the two fluids; CTxI (*P *= 0.0002), NTx (*P *< 0.0001), osteocalcin (*P *= 0.0002) and MMP-3 (*P *= 0.038) (Table 2). These data strongly suggest a signal knee source of these biomarkers and the ability of a serum concentration to reflect early signal knee events following acute injury. Of note, the acute phase reactant protein, CRP, demonstrated the reverse pattern; CRP concentrations in serum exceeded those in synovial fluid and there was a significant correlation (*P *= 0.002) between the serum and synovial fluid concentration compatible with passive diffusion of CRP into the synovial fluid, suggesting a systemic response to acute injury rather than a signal knee specific response. In the acute injury phase, the duration and/or level of elevation of CRP in the serum may be a useful indicator of extent and severity of tissue injury but this would require correlation with arthroscopic or MRI assessments of joint inflammation and injury.

## Discussion

It is important to understand the amount and type of joint damage after acute injury to identify novel therapeutic strategies that could be employed to better treat the symptoms and reduce the future risk of post-traumatic OA development. In this study we investigated the changes in biomarker concentrations at the time of sports-related acute knee injury and after a short period of recovery prior to surgery in 11 young patients with a mean age of 23 years. The patients were all part of a small randomized placebo-controlled pilot study of IL-Ra to determine the feasibility and establish the methodology for future clinical trials in acute joint injury. The initial samples were obtained prior to IL-1Ra injection and the second set of samples was obtained a mean four weeks later. No statistical difference was observed between the treatment and placebo arms for any of the biomarkers presented in this paper although we acknowledge that a sample of this size may have limited power to detect such differences. However, we believe it is reasonable to expect that these effects on the biomarker levels would be minimal because a single dose of IL-1Ra has a very short *in vivo *half-life, estimated at four to six hours after bolus subcutaneous injection [25]. We investigated a total of 21 different biomarkers representing a wide range of joint relevant molecules in both synovial fluid and serum to better understand the changes which occur after acute injury and to identify biomarkers which might be most useful for following the effectiveness of future disease modifying protocols.

The investigation of several different classes of biomarkers after acute injury demonstrated that the proteoglycan biomarkers (_sf_GAG, _sf_ARGS aggrecan and _sf_FA846) decreased with time from injury which mirrored the inflammation biomarker _sf_CRP and _sf_lubricin. A decreasing trend was also observed for _sf_COMP, while interestingly _sf_MMP-3 did not change over time. In contrast, all the collagen and bone biomarkers increased with time from injury. Taken together, these results demonstrate that the cartilage within the injured joint responds to the initial acute injury with a wave of proteoglycan and non-collagenous protein loss. However, as the concentrations of proteoglycans and small cartilage molecules declined, there was apparent onset of collagen damage with a rise in synovial fluid collagen biomarkers. These *in vivo *findings after acute injury are analogous to the temporal patterns of matrix epitope release *in vitro *from cartilage explants stimulated with proinflammatory cytokines in which there is an initial loss of proteoglycan followed by collagen loss [26]. It has long been known from animal studies [27,28] that the loss of proteoglycan is reversible while once the collagen is lost, the cartilage is irreparably damaged [29]. Since these data show significant cartilage collagen epitope loss *in vivo *within even the first month after injury, it would appear that critical and possibly irreversible damage is sustained within weeks of severe knee injury with an ACL tear. These data suggest the possible need for very early intervention to prevent post-traumatic-osteoarthritis. More detailed studies are required to better characterize these early changes and to link them to the future risk of post-traumatic OA known from previous studies to be 50% by 10 to 15 years following ACL injury [1,3].

The most likely cause of this observed early cartilage and joint damage after acute injury is through the effect of pro-inflammatory cytokines released into the synovial fluid. This observed inflammatory driven damage matches that observed in pro-inflammatory cytokine-induced cartilage degeneration in explant models [26] and the catabolic processes observed in rheumatoid arthritis and preclinical animal models of OA. These observations suggest that some of the already existing small molecules that have demonstrated chondroprotective effects *in vitro*, in animal models and in humans with rheumatoid arthritis may constitute new treatment strategies to prevent the irreversible cartilage damage in the form of collagen loss after acute injury and thereby offer the hope of preventing future onset of injury related OA. These include: the biologic anti-cytokine therapies directed towards IL-1α, TNFα or IL-6 that are successful rheumatoid arthritis therapies [30]; p38 mitogen activated protein kinases (MAPK) pathway inhibitors, protective in a rat model of OA [31,32] and in explant culture [33]; statins that inhibit cartilage breakdown by reducing protein prenylation [34,35]; and sulfazalazine that can inhibit collagenase production by inhibiting the NFkB pathway in cartilage explant models [36]. Direct inhibition of catabolic enzymes may also be a viable short term target for cartilage and joint protection after acute injury. Inhibition of proteoglycan loss has been shown to be effective at preventing OA in animals [37-39] while inhibition of the collagen degrading collagenase enzymes can protect cartilage from irreversible collagen loss [40]. Any of these potential therapies could be administered early after acute injury for a short period of time to prevent the initial and potentially irreparable cartilage and joint damage which occurs after acute injury.

In this study we also compared the concentrations of different biomarkers in the synovial fluid and the serum to determine how the serum concentrations reflect the changes in the signal knee. These data are important for future clinical trials as measuring biomarkers in the serum is easier than obtaining serial synovial fluid samples. Only MMP-3 and the bone biomarkers, CTxI, NTx and osteocalcin demonstrated a significant correlation between serum and synovial fluid concentrations suggesting that these serum biomarkers may accurately reflect the activity in the signal knee. The lack of correlation between the serum and synovial fluid concentrations for many of the standard biomarkers may be due to their rapid clearance from the joint or systemic circulation, or significant confounding of serum concentrations by other sources of biomarker epitopes. While there has been much research using these biomarkers (see comprehensive recent reviews by van Spil *et al*. [41] and Kraus *et al*. [42]), more research is required to better profile both the currently available biomarkers and to develop more specific biomarkers which are less sensitive to systemic contributions.

## Conclusions

The results presented in this manuscript demonstrate that there is significant and measurable cartilage and bone damage after acute knee injury. We also noted that the release of biomarkers from cartilage matches the profile observed from *in vitro *cartilage explants models treated with pro-inflammatory cytokines. This similarity between the damage which occurs in acute injury and the cartilage explant models suggests that much of the chondroprotective research performed in cartilage explant systems could be directly translated to the treatment of acute injury to reduce or even prevent early cartilage damage, thereby potentially reducing the risk of later early onset OA.

## Abbreviations

ADAMTS: A Disintegrin And Metalloprotease with ThromboSpondin motifs; ACL: anterior cruciate ligament; ANOVA: ANOVA Analysis of Variance; ARGS: Alanine-Arginine-Glycine-Serine; Asx, *a*spartate and asparagine; COMP: cartilage oligomeric matrix protein; CRP: C-Reactive protein; CTxI: C-terminal crosslinked telopeptide type I collagen; _sf_CTxII: C-terminal crosslinked telopeptide type II collagen; GAG: glycosaminoglycan; HPLC: high performance liquid chromatography; HRP: horse radish peroxidase; IL: interleukin; IL-1Ra: IL-1 receptor antagonist; mAb: monoclonal antibody; MAPK: mitogen activated protein kinase; MMP: matrix metalloproteinase; MRI: magnetic resonance imaging; _sf_NTx: N-terminal telopeptides of type I collagen; OA: osteoarthritis; RT: room temperature; TIMP: tissue inhibitor of metalloproteinase; TMB: 3,3', 5,5"-tetramethylbenzidine; TNF: tumor necrosis factor; v/v: volume/volume.

## Competing interests

JC, TS and VBK declare that they have no competing interests. CRF is an employee of and holds stock/stock options in Pfizer.

## Authors' contributions

JC, TS and CRF performed and/or coordinated biomarker analyses. JC performed statistical analyses and drafted the manuscript. VBK designed and supervised all aspects of the study. All authors participated in manuscript editing. All authors read and approved the final manuscript.
